# Evolution and expression of the fructokinase gene family in *Saccharum*

**DOI:** 10.1186/s12864-017-3535-7

**Published:** 2017-02-21

**Authors:** Yihong Chen, Qing Zhang, Weichang Hu, Xingtan Zhang, Liming Wang, Xiuting Hua, Qingyi Yu, Ray Ming, Jisen Zhang

**Affiliations:** 1 Center for Genomics and Biotechnology, Fujian Provincial Key Laboratory of Haixia Applied Plant Systems Biology, Haixia Institute of Science and Technology (HIST), Fujian Agriculture and Forestry University, Fuzhou, 350002 China; 2 College of Life Sciences, Fujian Normal University, Fuzhou, 350117 China; 3 Key Laboratory of Ministry of Education for Genetics, Breeding and Multiple Utilization of Crops, Fujian Agriculture and Forestry University, Fuzhou, 350002 China; 4 Texas A&M AgriLife Research, Department of Plant Pathology and Microbiology, Texas A&M University System, Dallas, TX 75252 USA; 5 Department of Plant Biology, University of Illinois at Urbana-Champaign, Urbana, IL 61801 USA

**Keywords:** Fructokinase gene family, *Saccharum*, Gene expression, Gene evolution, Polyploidy

## Abstract

**Background:**

Sugarcane is an important sugar crop contributing up to about 80% of the world sugar production. Efforts to characterize the genes involved in sugar metabolism at the molecular level are growing since increasing sugar content is a major goal in the breeding of new sugarcane varieties. Fructokinases (FRK) are the main fructose phosphorylating enzymes with high substrate specificity and affinity.

**Results:**

In this study, by combining comparative genomics approaches with BAC resources, seven fructokinase genes were identified in *S. spontaneum*. Phylogenetic analysis based on representative monocotyledon and dicotyledon plant species suggested that the FRK gene family is ancient and its evolutionary history can be traced in duplicated descending order: *SsFRK4, SsFRK6*/*SsFRK7,SsFRK5*, *SsFRK3* and *SsFRK1*/*SsFRK2*. Among the close orthologs, the number and position of exons in FRKs were conserved; in contrast, the size of introns varied among the paralogous FRKs in *Saccharum. G*enomic constraints were analyzed within the gene alleles and between *S. spontaneum* and *Sorghum bicolor*, and gene expression analysis was performed under drought stress and with exogenous applications of plant hormones. *FRK1*, which was under strong functional constraint selection, was conserved among the gene allelic haplotypes, and displayed dominant expression levels among the gene families in the control conditions, suggesting that *FRK1* plays a major role in the phosphorylation of fructose. *FRK3* and *FRK5* were dramatically induced under drought stress, and *FRK5* was also found to increase its expression levels in the mature stage of *Saccharum*. Similarly, *FRK3* and *FRK5* were induced in response to drought stress in *Saccharum. FRK2* and *FRK7* displayed lower expression levels than the other *FRK* family members; *FRK2* was under strong genomic selection constraints whereas *FRK7* was under neutral selection*. FRK7* may have become functionally redundant in *Saccharum* through pseudogenization*. FRK4* and *FRK6* shared the most similar expression pattern: *FRK4* was revealed to have higher expression levels in mature tissues than in premature tissues of *Saccharum*, and *FRK6* presented a slight increase under drought stress.

**Conclusions:**

Our study presents a comprehensive genomic study of the entire FRK gene family in *Saccharum*, providing the foundations for approaches to characterize the molecular mechanism regulated by the *SsFRK* family in sugarcane.

**Electronic supplementary material:**

The online version of this article (doi:10.1186/s12864-017-3535-7) contains supplementary material, which is available to authorized users.

## Background

Sugarcane (*Saccharum spp.)* is a large, perennial, tropical or subtropical crop. It is one of the world’s most produced crops (FAOSTAT, 2015) contributing about 80% of the world sugar production (FAOSTAT, 2010). Sugarcane also plays an increasingly important role in the biofuel field accounting for 40% of ethanol production worldwide [1]. Sugarcane mature stalks contain about 9 to 18% sucrose [2]. The genus *Saccharum* includes two wild species, *S. spontaneum* and *S. robustum,* and one cultivar species *S. officinarum*; these three species are thought to be the founding species. Majority of both *S. robustum* and *S. officinarum* are octoploid, with a basic chromosome number x = 8, whereas *S. spontaneum*’s chromosome number ranges from 2n = 36 to 128 with the majority of basic chromosome number x = 10 [3–5]. *S. officinarum* is a high-glucose species with, and is thought to be derived from *S. robustum* as these two species have the same center of diversity in New Guinea [6]. Modern sugarcane cultivars are hybrids derived from the cross between *S. officinarum* and *S. spontaneum,* resulting in extreme allopolyploidy levels that can range from octoploidy (x = 8) to dodecaploidy (x = 12). Most of the basic molecular and genetic analyses remain inconclusive in sugarcane due to the lack of genomic information of this complex genome. Typically, expressed sequence tags (EST) resources are used for sugarcane gene and gene family discovery, and these were the sole resource prior to the release of sorghum genome [7, 8]. Sorghum is the closest diploid species of *Saccharum,* and has a genome size about 760 Mb which has been sequenced [9]. Comparative genomic studies demonstrated that sorghum has a small, diploid genome that contains fewer chromosomal rearrangements and shares a strong collinearity with sugarcane, thus providing the best reference model system to study sugarcane genomics [10–13]. It is possible to identify sugarcane genes by combining the BAC sequences and the available sorghum genome data.

The study of sugar metabolism in sugarcane is one of the most active areas of research. In plants, sucrose is cleaved into UDG-glucose and fructose by sucrose synthase (SUS), or can be cleaved into glucose and fructose by invertases for further sugar metabolism [14]. Fructokinases (FRK, EC 2.7.1.4) can phosphorylate free fructose with high substrate specificity and affinity [15]. Hence, the phosphorylation of fructose by FRKs is believed to be necessary for sucrose cleavage and sugar metabolism, both of which are essential for proper development of vascular tissue [16, 17]. To date, plant FRKs have been purified from several plants, such as *Arabidopsis* [18, 19], sugar beet [20], potato [21], tomato [22, 23], rice [24], soybean [25], maize [26], pea seeds [27]. Two isoforms of sugarcane fructokinases, FRK1 and FRK2 were purified from the culm of sugarcane [28]. Nevertheless, due to the complex sugarcane genetic background, identification of sugarcane FRK genes is still unavailable.

Understanding the molecular structure and evolution of a gene family is a key step towards the understanding of the physiological roles, metabolic mechanism and potential function of its members, the necessary groundwork for possible future transgenic studies. In this study, to characterize the gene evolution and possible functions of the FRK gene family in sugarcane, we performed gene family identification based on the combination of comparative genomics strategies and high genome coverage of bacterial artificial chromosomes (BACs) library resources, and investigated gene expression levels by RNA-seq and RT-qPCR. The analysis of this study mainly focuses on 1) the evolutionary relationship and gene structure of the FRK gene families, and 2) characterization of the gene expression patterns of the FRK gene family to predict function.

## Results

### Identification of seven FRK genes in *S. spontaneum*

Based on comparative genomics, seven well annotated sorghum FRK genes, referred to as *SbFRKs*, were identified (Table 1). These seven *SbFRKs* were located in chr01, chr03, chr07, chr09 and chr10 of sorghum genome. None of these seven was observed to have undergone tandem duplication. Using these well-annotated *SbFRK* genes as references to design probes (Table 1), 12 BAC sequences for *FRKs* were screened and sequenced from *S. spontaneum*. Further analysis revealed that the 12 *S. spontaneum* sequences encoded seven *FRKs* referred to as *SsFRK1-SsFRK7*. In these 12 sequences, *SsFRK1* had three allelic haplotypes, *SsFRK2, SsFRK3,* and *SsFRK5* had two allelic haplotypes, *SsFRK4, SsFRK6* and *SsFRK7* only had one allele. The allelic haplotypes of each FRK are indicated by an additional “-h1” to “-h3” at the end of gene name. Using the gene model sequences of the annotated FRK genes as queries, an in-house EST and Genbank database were extensively searched. The results showed that six of the *SsFRKs* had the corresponding ESTs in the Genbank database except *SsFRK3* (Additional file 1).

**Table 1:** Information of putative FRK genes in sorghum

Corresponding gene name in sugarcane	Gene ID	Transcript ID	Protein ID	Location of the gene
*SsFRK1*	*Sb03g042460*	XM_002458864.1	XP_002458909.1	NC_012872.1|:c69874432-69871609 chromosome 3
*SsFRK2*	*Sb10g008280*	XM_002436715.1	XP_002436760.1	NC_012879.1|:8422480–8426810 chromosome 10
*SsFRK3*	*Sb01g015030*	XM_002466795.1	XP_002466840.1	NC_012870.1|:c14441197–14437165 chromosome 1
*SsFRK4*	*Sb01g046230*	XM_002468410.1	XP_002468455.1	NC_012870.1|:c69274783–69271917 chromosome 1
*SsFRK5*	*Sb03g040010*	XM_002456599.1	XP_002456644.1	NC_012872.1|:67626538–67628943 chromosome 3
*SsFRK6*	*Sb09g018040*	XM_002440925.1	XP_002440970.1	NC_012878.1|:c45125278–45122178 chromosome 9
*SsFRK7*	*Sb07g027900*	XM_002445866.1	XP_002445911.1	NC_012876.1|:c62873182–62869987 chromosome 7

**Fig. 1:**
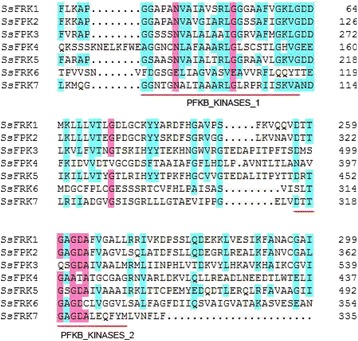
Conserved domains of the SsFRKs gene family. Two conserved domains, pfkb1 and pfkb2, of FRKs were identified using BLASTp (http://blast.ncbi.nlm.nih.gov/Blast.cgi) and InterPro (http://www.ebi.ac.uk/interpro/scan.html) with protein sequences

The coding sequences of *FRKs* were translated into protein sequences. The alignment of *SsFRKs* revealed that *SsFRKs* harbors two conserved domains, pfkB1 [AG]-G-x(0,1)-[GAP]-x-N-{AGLS}-[STA]-x(2)-{A}-x-{G}-{GNKA}-[GS]-x(9)-G;and pfkB2:[DNSK]-[PSTV]-x-[SAG](2)-[GD]-D-x(3)-[SAGV]-[AG]-[LIVMFYA]-[LIVMSTAP] (Fig. 1), demonstrating that these sequences belong to FRK family members. The molecular weights of the SsFRKs ranged between 34.7 and 66.2 kDa, and three FRKs (FRK3, FRK4, FRK5) had a larger size than the others (Table 2). Comparative analysis between sorghum and *S. spontanenum* were performed to investigate the divergence of *SsFRKs/SbFRKs.* The results showed that the *SsFRKs* have molecular weights that are similar to their orthologous FRK in sorghum, except for *FRK6* and *FRK7*. Consistently, *SsFRKs* shared high identities (> = 93%) with their orthologous *SbFRKs*, well above those of *FRK6* (77%) and *FRK7* (91%). These results indicated that *FRK6* and *FRK7* had undergone stronger evolutionary dynamics after the split of *Saccharum* and *Sorghum*.

**Table 2:** Comparison of the characterization of the FRKs between sugarcane and sorghum

Sorghum					Sugarcane					
Gene name	Protein size	Mw (kD)	Domains	I﻿soelectric point(pI)	Gene name	Protein size	Mw (kD)	Domains	Isoelectric point(pI)	Identity
*SbFRK1*	323	34.7	PfkB	5.00	*SsFRK1*	323	34.7	PfkB	4.87	99%
*SbFRK2*	388	41.2	PfkB	5.81	*SsFRK2*	388	41.2	PfkB	5.56	97%
*SbFRK3*	594	66.2	PfkB	7.23	*SsFRK3*	583	65.0	PfkB	6.44	94%
*SbFRK4*	488	52.2	PfkB	5.83	*SsFRK4*	481	51.4	PfkB	6.09	93%
*SbFRK5*	522	58.7	PfkB	6.36	*SsFRK5*	524	58.9	PfkB	6.03	97%
*SbFRK6*	381	42.0	PfkB	5.33	*SsFRK6*	302	32.8	PfkB	5.43	91%
*SbFRK7*	335	36.1	PfkB	9.14	*SsFRK7*	364	38.2	PfkB	4.95	77%

To analyze the divergence among the paralogous *FRKs* in *S. spontaneum*, an intercomparison of protein sequences among *SsFRKs* was performed (Additional file 2). Results showed that *SsFRKs* shared protein sequence similarities ranging between 25 and 64%. Among the *SsFRKs, SsFRK1* and *SsFRK2* displayed the highest similarity, 64%, whereas the other pairwise similarities were under 40%, and most of the 14 of the analyzed gene pairs were excluded from the comparison results due to the high sequence divergence among the gene families, suggesting that the *SsFRK* is an ancient gene family with potentially high functional divergence.

### Comparative analysis of SsFRK allelic haplotypes

**Fig. 2:**
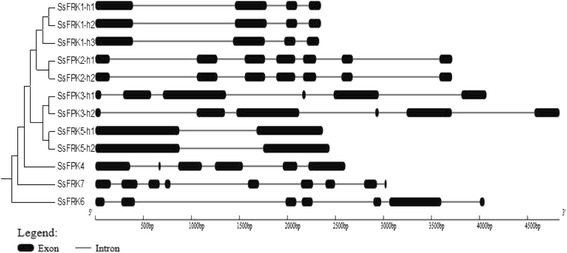
Comparison of the allelic gene structure of SsFRKs. The allelic haplotypes of each FRK was indicated additional “-h1” to “-h3” at the end of gene name. Boxes represent exons

**Fig. 3:**
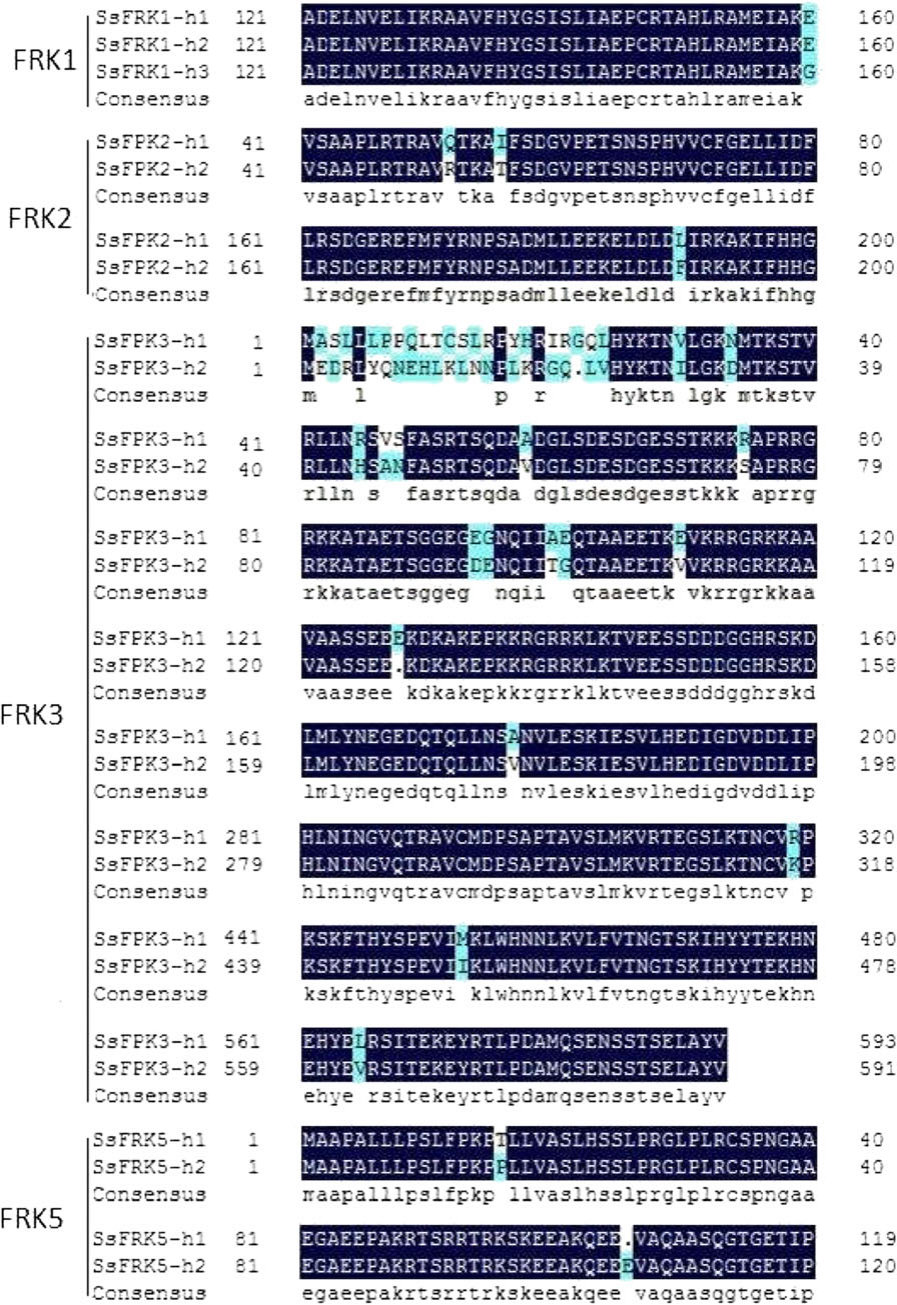
Alignment of the amino acid sequences of SsFRK haplotypes. Amino acid sequences of haplotypes were aligned using the DNAMAN program. The difference between haplotypes is highlighted in blue

Of the four *SsFRKs* with allelic haplotype sequences, three *SsFRKs* (*SsFRK1, SsFRK2* and *SsFRK5*) shared very high similarity with identity higher than 99%, and *SsFRK3* was less constraint within its alleles of 93.33% for genomic sequences and 95.58% for protein sequences. Similarly, gene structure comparisons within the allelic haplotype of the four genes also demonstrated that the allelic haplotype of three *SsFRKs* (*SsFRK1, SsFRK2* and *SsFRK5*) were highly conserved, while *SsFRK3* alleles had a deletion/insertion for their first intron (Fig. 2). Protein sequence alignment of the allelic haplotypes showed that SsFRK1, SsFRK2, SsFRK3 and SsFRK5 had 1, 3, 37 and 2 variant amino acids, respectively (Table 3 and Fig. 3). SsFRK3 presented great variation at the N terminal (Fig. 3). However, none of these variable amino acids located to the two pfrk domains, indicating functional conservation among these allelic haplotypes.

**Table 3:** The variation of deduced amino acid sequences among allelic haplotypes within the four SsFRK

FRK	No. of variations (SsFRK-h1 vs other haplotype)	Amino acid variations
*SsFRK1*	1	E160G
*SsFRK2*	3	Q12R,I16T,L190F
*SsFRK3*	37	A2E,S3D,L4R,L6Y,P7Q,P8N,Q9E,L10H,T11L,C12K,S13L,L14N,R15N,Y17L,H18K,I20G,R21Q,G22-insertion,Q23L,L24V,V30I,N34D,R45H,V47A,S48N,A58V,R75S,E94D,G95E,A100T,E101G,E110V,E128-insertion,A177V,R319K,M453I,L565V
*SsFRK4*	N/A	
*SsFRK5*	2	T16P,106E-deletion
*SsFRK6*	N/A	
*SsFRK7*	N/A

### The evolutionary function constraint in SsFRK families

**Fig. 4:**
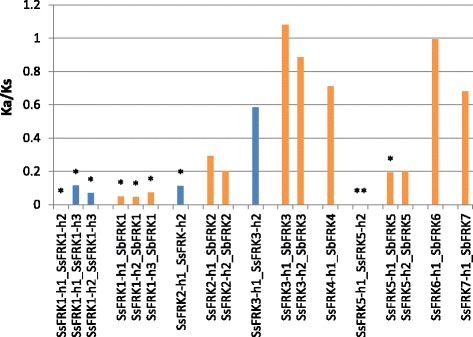
The Ka/Ks of SsFRK haplotypes and SsFRK-SbFRK. The lower value of Ka/Ks is indicated by stars. The blue color indicates the Ka/Ks of gene pair allele comparisons, orange color indicates the Ka/Ks of orthologs genes between sorghum and *S. spontaneum*

The evolutionary function constraint was evaluated for allelic haplotypes based on nonsynonymous to synonymous substitution ratio (*Ka/Ks*) (Fig. 4). The results revealed that *SsFRK1, SsFRK2* and *SsFRK5* were under strong purifying selection as the *Ka/Ks* ratio was lower than 0.3, while *FRK3* was approximately under neutral selection (pressure) with a *Ka/Ks* ratio of 0.6. In addition, the *Ka/Ks* ratio of pairwise of *S.spontaneum* - *sorghum* showed that *SsFRK4, SsFRK6,* and *SsFRK7* have a larger value above 0.6. These results showed that different *SsFRKs* had undergone different evolutionary forces.

### Phylogenetic analysis of *SsFRKs* and other plant *FRKs*

**Fig. 5:**
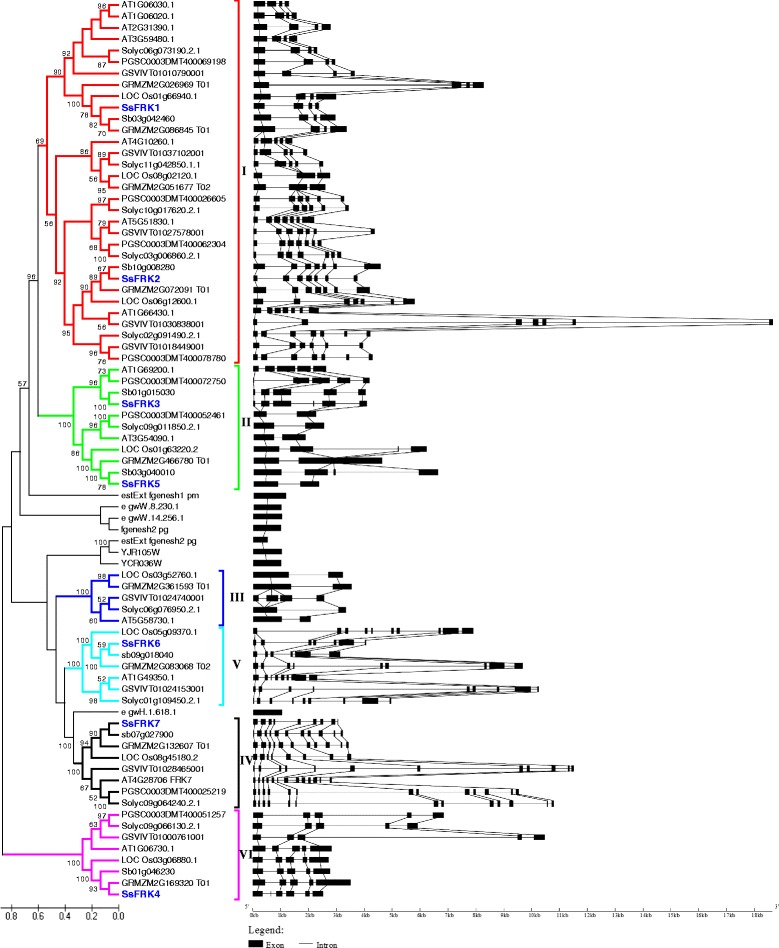
Comparison of gene structure and phylogenetic analysis of the eight members of SsFRK gene family. Unrooted phylogenetic tree of plant FRK proteins constructed using the neighbour-joining method with MEGA 5.2.1 program. GRMZM2G: *Zea mays*, Sb: *Sorghum bicolor*, LOC_Os: *Oryza sativa*, SsFEK: *Saccharum spp*, At: *Arabidopsis thaliana*, GSVIVT: *Vitis vinifera*, Solyc: *Solanum lycopersicum*, PGSC: *Solanum tuberosum.* FRK gene families are also shown for comparison. Boxes represent exons, triangles represent transposons. The star signs indicates potential gene duplication events for the *SsFRK* families

To analyze the phylogenetics of the FRK gene family, 71 *FRKs* were selected from eight representative plant species including four monocotyledons (sugarcane, sorghum, rice, and maize), and four dicotyledons (potato, *Arabidopsis*, grape, and tomato) and a phylogenetic tree was constructed using the ClustalX with Neighbor-Joining method (Fig. 5). The *FRKs* were phylogenetically distributed into six groups, referred to as group I - group VI. Each of the groups contained FRK genes from both monocotyledons and dicotyledons, suggesting that the six gene groups originated before the monocot-dicot split. In group V, the dicot and monocot genes were separated into two subgroups, indicating that the genes in this group diverged from an evolutionary event after the common ancestor of dicots and monocots. Group I is the largest group, counting 32 *FRKs* including *SsFRK1* and *SsFRK2*. In this group, genes were distributed into two subgroups, each of which could be further divides into two subgroups with one consisting of monocot specific genes, and the other containing both dicot and monocot genes, suggesting that gene expansion occurred in dicot species before the divergence of dicots and monocots. Group II contained 11 *FRKs* including *SsFRK3* and *SsFRK5* from seven plant species except grape. Group III consists of 5 *FRKs* from 5 of the 8 plant species (excluding sugarcane, sorghum and potato), and it is likely that gene loss is a recent event (after the origin of *Trib. Andropogoneae Dumort* in monocots) that occurred in both of dicot and monocot plants. Both group IV and VI contain 8 *FRKs* with one from each the eight plant species, revealing that the genes in two groups shared an ancient ancestor before the divergence of dicots and monocots. Group V contained 7 *FRKs* from 7 of the 8 plant species (excluding potato), suggesting that a recent *FRK* gene loss event occurred after the split of potato and tomato in *Solanum*.

The *FRKs of Chlamydomonas* and yeast were used as the out group for the phylogenetic analysis of the origin of plant FRKs (Fig. 5). Group I and Group II were phylogenetically clustered together with an out group containing the FRKs of *Chlamydomonas* and yeast, whereas, group III, V and VI were clustered with different FRKs of *Chlamydomonas* and yeast. These results suggested that plant FRKs originated before the divergence of lower eukaryotes (such as *Chlamydomonas* and yeast) and land plants. Moreover, group VI were independently grouped together with a yeast FRK, indicating that a remote gene duplication event occurred (for the origin of O-II and O-III) before the split of yeast and land plants. In addition, group IV FRKs were separated from the other 5 groups and have not grouped with the lower eukaryotes gene, indicating that FRKs in this group are specific to land plants.

### Exon/Intron organization of the *SsFRKs* family and other plant *FRKs*

In the examined plant species, the gene structures of *FRK* were variable in both exon number and size. In group I, the exon number ranged from three to seven, while, the genes of each subgroup in this group contained similar exon numbers. In group II, the *FRKs* had exon numbers ranging from 2 to 6, similarly genes in each of the subgroups had a similar exon number except for *SsFRK3,* which had 1 more exons. These results demonstrated that the exon/intron reorganization of *FRKs* is a recent evolutionary event that occurred after the split of dicots and moncots. Group III had two exons beside the corresponding genes of the grape in which the first exon was split in two yielding a total of four exons and not three. In group IV, *FRKs* had five exons except *SsFRK4* which harbors one additional exon originating from exonization in its corresponding first intron. In groups V and VI, *FRKs* harbored more and smaller exons than the genes in the other groups. Also, the gene sizes of the *FRKs* varied among the genes in these two groups due to intron expansion. Moreover, the FRKs in grape were found to have longer introns than other examined species, which is consistent with the results of whole genome analysis for grape genes [29].

The SsFRKs genes display great variation in exon numbers which range from 2 to 9, with the introns aligning in accordance to the GT-AG rule for splicing sites (Fig. 2 and Fig. 5). Both *SsFRK3* alleles contained one additional exon compared to its ortholog in sorghum, causing an exonization event in its third intron, indicating the gene restructured after the divergence of *Saccharum* and *Sorghum* and before whole genome duplication in *S. spontaneum*. Similarly, *SsFRK4* harbored one additional exon compared to its ortholog in sorghum. These findings present evidence that the recent whole genome duplication of *S. spontaneum* provided the evolutionary forces for the restructuring of the SsFRK genes.

### Gene expression of *SsFRKs* among *Saccharum* species and *Saccharum* hybrid

The gene identification makes it possible to investigate gene expression to evaluate the potential function of the gene families. We performed comparative transcriptome profiling among three *Saccharum* species and the *Saccharum* hybrid at different developmental stages of seedlings with different plant hormones and five different tissues from the mature leaf (mature and leaf roll) and stalks (mature, maturing and immature) by RNA-seq method. The Reads Per Kilobases per Million reads (RPKM) value of the examined genes were verified by q-RT-PCR in three tissue types from two *Saccharum* species, *S. officinarum* and *S. spontaneum.* The results were positively correlated with RPKM values (Additional file 3).

**Fig. 6:**
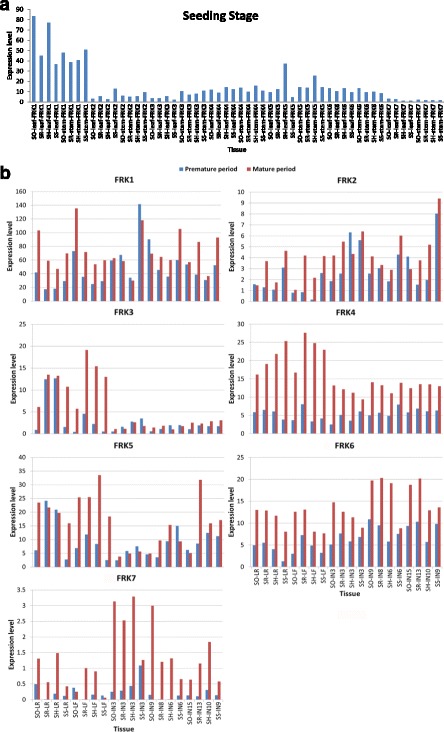
**a** Expression levels of SsFRK gene family members at the seedling stage. SR: *S. robustum* Molokai6081; SS: *S. spontaneum* SES208; SO: *S. officinarum* LA Purple; SH: hybrid cultivar ROC-22. The gene expression level was based on RPKM value. **b** Expression levels of SsFRKs gene family members in pre-mature stage and mature stage tissues. SR: *S. robustum* Molokai6081; SS: *S. spontaneum* SES208; SO: *S. officinarum* LA Purple; SH: hybrid cultivar ROC-22; IN, internode; LR, leaf roll; LF, leaf. Internodes 3,9,15, internodes 3,9,15, internodes 3,8,13 and internodes 3, 6, 9 were from *Saccharum officinarum* (LA Purple), ROC-22, *Saccharum robustum* (Molokai6081) and *Saccharum spontaneum* (SES208), respectively. The gene expression level was based on RPKM value

In all examined tissues, *SsFRK1* was the most abundantly expressed gene among the *SsFRK* family, *SsFRK7* presented the lowest expression levels (Fig. 6), and the remaining genes showed similar expression levels. These results suggested that *SsFRK1* was the dominant member of the gene family.

At the seedling stage, expression levels of *FRK1s* were higher in both *S.officinarum* and the *Saccharum* hybrid than in the other two species, which may be caused by that these two *Saccharum* plants had similar genetic background. Similar expression levels of *SsFRKs* were observed in the leaves and the stems, except the *FRK3’*s expression that was higher in the stem than in leaves. At the premature stage (7 months old), the expression of *FRK3* was observed to decrease in comparison to seedling stages. In general, *FRKs* had higher expression levels in the mature period than in the premature period, suggesting that *FRKs* are involved in sugarcane sugar accumulation as mature plants contained higher sugar contents than the premature plants. The most abundantly expressed gene, *FRK1*, was observed to increase its expression levels in all analyzed leaf tissue including the leaf roll but decreased after the mature stage in some stem tissue (internode 3 of *Saccharum* hybrids and *S. spontaneum*, and internode 9 of *S. officinarum*), indicating that *FRK1* is more active in the sugar metabolism of source tissues than in that of sink tissues. Similarly, *FRK3* had higher expression levels in the source tissues than the sink tissues.

Polyethylene glycol (PEG) can be used to modify the osmotic potential of the nutrient solution culture and thus induce plant water deficit [30]. The accumulation of soluble sugars in response to drought stress has a positive correlation with the increase of leaf water contents [31–33]. In the leaves of seedling with PEG stress, the expression of *FRK1* was suppressed in three of the examined *Saccharum* species (*S. robustum, S. officinarum* and hybrid cultivar (ROC-22) except *S. spontaneum),* where *FRK1*’s expression was induced, *FRK2*’s expression was not altered, and expression of the remaining five genes *(FRK3, FKR4, FRK5, FRK6* and *FRK7*) increased. It was unexpected to observe that expression of *FRK3* and *FRK5* dramatically increased under PEG treatment, and had similar or higher mRNA levels than *FRK1*. These results strongly suggested that phosphorylation of fructose contributes to the sugar accumulation under PEG-induced drought stress, and both *FRK3* and *FKR5* were the main players in response to PEG stress.

Plant growth and development depend on the phytohormone-mediated regulation of gene expression. In this study, we analyzed the gene expression in the leaves of seedlings of *Saccharum* plants treated with abscisic acid (ABA), auxin (IAA), ethephon (Et), or gibberellins (GA). Gene expression levels were altered in response to ABA treatment, especially for *SsFRK1, SsFRK3* and *SsFRK5*. Moreover, the gene expression of different FRKs varied in response to different ABA treatment times. For example, *SsFRK1* showed a tendency to increase expression levels, whereas *SsFRK5* decreased its expression level as the ABA treatment time was increased. No common shared gene expression change patterns could be identified for the *FRK* genes, suggesting that the *FRK*s are not genes directly regulated by ABA. In contrast to the ABA treatment, the gene expression of *FRKs* was not noticeably affected by the IAA treatment in *Saccharum*, except for *FRK1,* which had a more than five-fold increase in expression in the stems of *S. officinarum* after 24 h IAA treatment. In the leaves, *SsFRK1* displayed a gradual decreasing trend at 24 h, 48 h and 96 h, but the trend was reversed in the stems. Under Et treatment, the expressions of *FRK5* was suppressed in the majority of the examined tissues. Interestingly, *FRK1* was suppressed in the leaves of the *Saccharum* hybrid and *S. spontaneum* for all three time points of Et treatment. As for the remaining 5 FRK genes, Et treatment did not generally cause a great variation in their expression levels. Similarly to the treatment of GA of *Saccharum* plants, *FRK5* expression was inhibited in the majority of examined tissues, and the transcript of *FRK1* was inhibited in the leaf tissue in response to GA treatment at 48 h and 96 h, but was induced in the stem tissues. These results demonstrated that *FRKs* were co-expressed under GA and Et treatments.

Cluster analysis of gene expression showed that the *FRK* family has a similar expression pattern in all experimental groups except for the GA treatment group, where *FRK7*’s expression pattern resembled that of *FRK2* instead of *FRK3* (Additional files 4, 5, 6, 7 and 8).

## Discussion

In sugarcane, sugar metabolism is assumed to be one of the most important pathways in crop science due to its role of sugar accumulation. FRK is one of the main enzymes phosphorylating fructose to fructose 6-phosphate (F6P). Despite its essential role in fructose metabolism, FRKs are still poorly understood due to the complex sugarcane genome. Genomics and expressional studies are the key step for further gene function and molecular breeding studies. The available of sorghum genome [34], and the BAC of *S. spontaneum* make it possible to identify the FRK gene family members in *Saccharum*.

### Gene evolution in the *FRK* family

In this study, seven *FRK*s were identified in the sorghum genome. Using sorghum *FRKs* as a reference, the same number of orthologous *FRK*s were identified in *S. spontaneum*. In comparison with previous studies on gene families in *Saccharum*, such as those on the phosphoenol pyruvate carboxylase gene, sucrose synthase [35], ATP-dependent phosphofructokinase [36], and sucrose transporters [37], SsFRKs presented higher divergence in their protein sequences (Additional file 2), indicating the *FRK*s are an ancient gene family. Phylogenetic analysis revealed that the FRK gene had undergone three rounds of gene duplication before the split of dicots and monocots (Fig. 5), further supporting the idea that *FRK*s are an ancient gene group.

Based on the phylogenetic analysis (Fig. 5), we can estimate the origin order of gene families. In the Ss*FRK* family, the last common ancestor (LCA) of *SsFRK* was suggested to have undergone gene duplication four times. *SsFRK4* was assumed to have originated from the first gene duplication, *SsFRK6/SsFRK7* from the second, *SsFRK3/ SsFRK5* from third, and the remaining two genes, *SsFRK1* and *SsFRK2,* from the fourth. Therefore, the evolutionary history of SsFRKs could be sorted by age in duplicated descending order: *SsFRK4, SsFRK6/SsFRK7, SsFRK3/ SsFRK5*, and *SsFRK1/SsFRK2.* Of the four gene duplication events, the related SsFRK of the first two gene duplications were clustered together with different out groups of FRKs, suggesting these two times duplications occurred before the split of land plants and *Chlamydomonas* more than 10 billion years ago. Thus, FRKs are belong to an ancestral gene family that existed before the origin of land plants.

Sorghum is the closest diploid plant species of *Saccharum*, and comparison of protein sequences between orthologues of *sorghum* and *S. spontaneum* may provide clues to understand the gene evolution of recent whole genome duplications in *Saccharum* after the split of Sorghum and *Saccharum*. Based on the phylogenetic analysis, *SsFRK1/SsFRK2* is suggested to be derived from the most recent duplication in this gene family, in addition to gene pairs of both *SsFRK1* and *SsFRK2* alleles were under strong selection constraints (Fig. 4), suggesting that these two genes were under functional constraint in *S. spontaneum*. Moreover, *SsFRK1/SbFRK1* were more conserved in their protein sequence (99%) than *SsFRK2/SbFRK2* (97%) (Table 2), Similarly, the gene structure and protein sequences were more conserved within the allelic haplotype of *SsFRK1* than that of *SsFRK2* (Fig. 2 and Table 3). Phylogenetic analysis may explain the differences of identities between these two recent duplication gene pairs in *Saccharum*. In the branch containing *SsFRK1*, *Trib. Andropogoneae Dumort* (*sorghum* and *S. spontaneum*) only has a single gene, while the other analyzed dicot plant species had at least two genes. But in the branch containing *SsFRK2*, all monocot plants only have one *FRK*. One of the FRKs in branch containing *SsFRK1* was lost after the divergence of *Trib. Andropogoneae Dumort* in Gramineae*,* and thus *SsFRK1* in *Trib. Andropogoneae Dumort may* replace the functional loss. Therefore, the *FRK1* was under a stronger functional constraint than *FRK2* in T*rib. Andropogoneae Dumort*.

*FRK3* was present in *Trib. Andropogoneae Dumort* but was absent from both rice and maize, indicating that the function of *FRK3* was replaceable in the Gramineae. *Ka/Ks* analysis suggesting that *FRK3* had a lower selective constraint than *FRK1, FRK4* and *FRK5* (Fig. 4). It is possible that *FRK3* is functionally redundant in *S. spontaneum*. Alternatively, *FRK3* may have been under positive selection, or demographic changes could have led to fixation of the most divergent allele in the *S. spontaneum* population.

*SsFRK7* was the only *SsFRK* clustered with its orthologs in *Chlamydomonas* in a branch (VI) (Fig. 4), moreover, the *SsFRK7/SbFRK7* had the lowest protein sequences similarity (77%) among the seven orthologous gene pairs. Land plants and and *Chlamydomonas* diverged over 1 billion years ago [38]*.* This phylogenetic results thus suggested that *SsFRK7* had undergone a different evolution compared to other *SsFRK*s (Fig. 5). *Ka/Ks* analysis revealed that *SsFRK7* had lower selective constraint than other *SsFRKs*. Furthermore, *SsFRK7* was distinct from the other *SsFRK*s for the protein sequence, and had the lowest gene expression levels among the *SsFRKs*. Therefore, the evolutionary mechanisms of *SsFRK7* could be caused by its functional redundancy in *S. spontaneum* since the long term divergence after the LCA of *SsFRK7* and the other *SsFRK*s.

### Gene expression and function in *Saccharum* plants

**Fig. 7:**
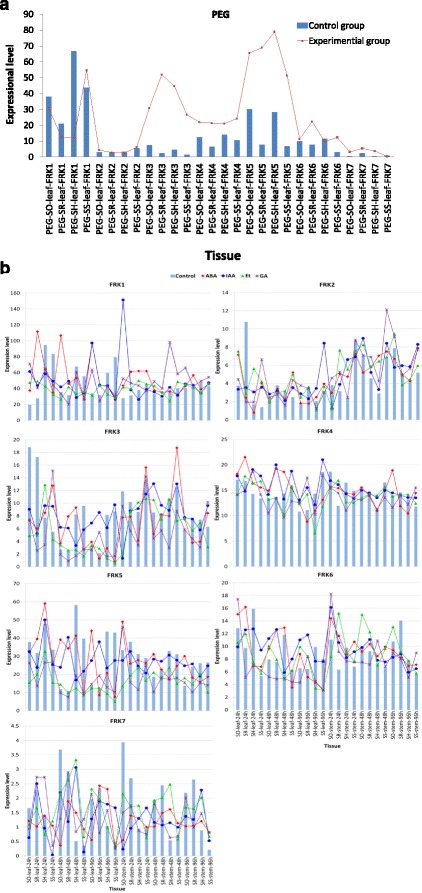
**a** Expression levels of SsFRK gene family members under PEG treatment. SR: *S. robustum* Molokai6081; SS: *S. spontaneum* SES208; SO: *S. officinarum* LA Purple; SH: hybrid cultivar ROC-22; The gene expression level was based on RPKM value. **b** Expression levels of the *SsFRK* gene family members under hormone treatment (ABA, IAA, Et and GA). SR: *S. robustum* Molokai6081; SS: *S. spontaneum* SES208; SO: *S. officinarum* LA Purple; SH: hybrid cultivar ROC-22; IN: internode; LR: leaf roll; LF: leaf. Internnodes 3,9,15, internnodes 3,9,15, internodes 3,8,13 and internodes 3, 6, 9 were from *Saccharum officinarum* (LA Purple), ROC-22, *Saccharum robustum* (Molokai6081) and *Saccharum spontaneum* (SES208), respectively. The gene expression level was based on RPKM value

Gene expression analysis could provide the first direct evidence to investigate gene function due to the challenges of engineering transgenic *Saccharum*. In this study, *SsFRK*s were found to be in the analyzed samples at widely different expression levels. *SsFRK1* presented the highest gene expression levels among the SsFRKs, suggesting *SsFRK1* is the key isoform involved in the phosphorylation of Fru to F6P, which may further explain the rationale that this gene was the most conserved among the *SsFRK*. However, under PEG-induced stress, the expression of *FRK1* was suppressed except in *S. spontaneum,* which had expression levels similar to the control. Both *FRK3* and *FRK5* were induced dramatically by PEG treatment, especially *FRK5*, which had higher gene expression levels than *FRK1*. Therefore, *FRK1* was found to be the key isoform under normal conditions but not the key player for in the responses to PEG stress. Moreover, with the treatment of plant hormones at standard concentrations, our results showed that *FRK1* had the predominant expression in the *FRKs* family (Figs. 6 and 7), suggesting that *FRK1* is the key isoform for plant growth and development. Phylogenetic analysis showed that *Solyc06g073190.2* was the closest orthologous gene of *SsFRK2* in tomato; this gene is the main *FRK* in tomato plants [16] and was further found to be important for vascular development [16, 17]. Vascular development is the foundation for sugar accumulation, pointing to the importance of *FRK1* in sugar accumulation in *Sacchaurm* in addition to sugar metabolism.

Both *FRK2* and *FRK7* had low expression levels in the examined tissues of the *Saccharum*, with *FRK7* having the lowest expression levels. These two genes did not present clear expression variations under PEG stress and hormone treatments (Figs. 6 and 7), suggesting that these two genes do not play a main role in fructose metabolism. However, *FRK2* was under strong selective constraint within the alleles and in comparison to sorghum, while *FRK7* was under neutral selection. Therefore, we suspected that *FRK2* had undergone a key functional division for the plant development, whereas *FRK7* was functionally redundant in *S. spontaneum*. The close ortholog of the *SsFRK2* gene in tomato (*Solyc03g006860.2.1*) is specifically expressed in the anthers during the late stages of pollen development and during pollen germination and has very low expression levels in the other tissues [39]. It is possible that *FRK2* is involved in the reproductive system of *Saccharum* as this *FRK* has low expression in the leaf and stem tissues. Further analysis of FRKs gene expression in the development of pollen and anthers would be necessary to investigate the potential function of *FRK2* in *Saccharum* plants.

*FRK3* and *FRK5* were both dramatically induced by PEG treatment, therefore, the three *FRKs* including *FRK1, FRK3* and *FRK5* were predominant expression under drought stress (Fig. 7a). These results revealed that *FRK3* and *FRK5* played a main role in response to drought stress in *Saccharum*, and indicated that *the* phosphorylation of Glc to G6P and Fru to F6P were involved in response to drought stress in *Saccharum.* In another main sugar crop, sugar beet *(Beta vulgaris)* roots, FRK activity was observed to increase in response to wound stress [40]. In sunflower (*Helianthus annuus*), under drought stress, a plastid *FRK* is co-upregulated with other genes related to carbon metabolism [41]. In maize, *FRK2* is upregulated in response to short-term salt stress [42]. In plants, *FRKs* are thought to be commonly involved in responses to abiotic stress. In contrast to *FRK3*, the expression levels of *FRK5* were generally suppressed by Et and GA In young premature *Saccharum* plants plants, but increased in more mature tissues. GAs generally promote plant growth and greatly influence plant stature and organ size, while Et regulates stress-related responses and/or growth retardation. Therefore, *FRK5* was suggested to play a role for plant growth in addition to stress tolerance response in *Saccharum.*

*FRK4* and *FRK6* had the closest expression profiles in most of the experiments from this study. Similarly to *FRK5*, the transcripts of *FRK4* increased in the mature tissues of *Saccharum* (Fig. 6b) and under PEG stress (Fig. 7a), supporting the idea that *FRKs* contribute to sugar accumulation and stress tolerance. Moreover, unlike *FRK5*, the gene expression of these two FRKs was not sensitive to the hormone treatments in the *Saccharum* plants. It was deduced that *FRK4* and *FRK6* were supposed to have different functional divisions to *FRK5* for plant growth and development. In addition, *FRK6* only presented a slight increase in its expression levels under PEG-induced drought stress, indicating that *FRK6* is not the major player in the response to drought stress in the *FRK* family in *Saccharum*.

Nevertheless, FRKs are considered to be the key enzymes for sugar metabolism in plants [16, 17]. In this study, we observed that *FRKs* generally have higher expression levels in mature tissues than in premature tissues (Fig. 6b), supporting the idea that FRK genes contribute to sugar accumulation as the mature tissues contained higher sugar content. However, in this study, there was no correlation between gene expression levels of *FRKs* and sugar content among the *Saccharum* species. Similarly, in our previous study for SUTs, no correlation was observed between gene expression levels of SUTs and sugar content in the *Saccharum* species [37]. These results suggest that the variation of sugar contents among the *Saccharum* species may not depend on the difference of gene expression of a small scales of sugar metabolism genes.

## Conclusions

In this study, by combining comparative genomics approaches with BACs, we identified the fructokinase gene family consisting of seven members in *S. spontaneum*. We performed a comprehensive analysis of the evolutionary genesis, gene alleles, phylogenetic relationships, and gene expression patterns for the identified FRKs in *Saccharum* plants. The results suggest that the FRK gene family is an ancient gene family with ancestral LCA, and their evolution history could be sorted by age in duplicated descending order: *SsFRK4, SsFRK6/SsFRK7, SsFRK3/ SsFRK5,* and *SsFRK1/SsFRK2.* Moreover, individual FRKs were analyzed. *FRK1* was under strong functional selection constraint and was conserved among the gene allelic haplotypes, in addition *FRK1* displaying the highest expression levels among the other *FRKs* under normal condition, suggesting that *FRK1* plays the leading role in phosphorylating Fru to F6P for plant development. *FRK3* and *FRK5* were identified as the key genes in response to drought stress. *FRK5* was likely play a prominent role in plant growth in addition to stress tolerance response in *Saccharum. FRK2* and *FRK7* had lower expression than the other gene members, whereas *FRK2* was under strong genomic selection constraint; *FRK7* was under neutral selection. *FRK2* may have undergone a functional division in *Saccharum* in agreement with the study of the *SsFRK2* ortholog in tomato. *FRK7* might be functionally redundant in *Saccharum* as a result of the process of pseudogenization. *FRK4* and *FRK6* had the closest expression pattern. *FRK4* was revealed to have higher expression levels in mature tissues than in premature tissues of *Saccharum*, supporting the hypothesis that this gene contributes to the sugar accumulation in *Saccharum* plants. *FRK6* presented slightly increase under drought stress. To investigate the function of these genes under stress, further experiments such as characterizing the spatio-temporal expression, enzyme activity assay, and targeted gene knock out technology like CRISPR-Cas9 system, would be necessary. This study may offer foundation work for the future study of the *SsFRK* gene family to characterize the physiological role and molecular mechanisms leading to sugar accumulation in sugarcane.

## Methods

### Plant materials

Two wild type *Saccharum* species, Molokai6081 (*S. robustum*, SR 2n = 8× = 80), and SES208 (*S. spontaneum*, SS, 2n = 8× = 64), one cultivated species, LA Purple (*S. officinarum*, SO, 2n = 8× = 80), and one hybrid cultivar ROC-22 (SH 2n = 8× = 100–130) were used in this study [43]. Plants were grown in the field on the campus of Fujian Agricultural and Forestry University (Fuzhou, China) in the February of 2015. Tissue samples from leaf roll, leaf, top internode (i.e., internode number 3), maturing internode (i.e., internode number 9 for ‘LA Purple and Roc-22, internode number 8 for Molokai6081, and internode number 6 for SES208) and mature internode (i.e., internode number 15 for ‘LA Purple’ and Roc-22), internode number 13 for Molokai6081, internode number 9 for SES208) were collected from premature 7-month-old sugarcane plants and 11-month-old mature sugarcane plants from different branches of the same individuals (as replicates). Internodes were numbered from the top, as previously described [44], and the corresponding internode number for the different *Saccharum* species, due to the variation in number of stems, was also established according to the previously described approach [45].

The plants for PEG and hormone treatment were grown in a growth chamber at 30 °C, 70% RH, and a 14 h:10 h L:D photoperiod. Seedlings were treated with PEG6000 (30%) for 48 h, and the leaf tissue was collected for RNA isolation. Seedlings were treated with gibberellin (GA,200 μM), abscisic acid(ABA, 200 μM), indole-3-acetic acid(IAA, 200 μM), or ethephon (Et, 200 μM) for 24, 48, and 96 h. Stem and leaf tissues from the seedlings of the four sugarcane species were collected from 35-day-old plants. Harvested tissue was immediately frozen in liquid nitrogen and stored at −80 °C prior to RNA isolation.

### Database search for the FRKs gene family

Due to the lack of reference information for whole FRK gene families in plant, we used the keyword “fructokinase” to search the model plant *Arabidopsis* genome database (TAIR 10 release, http://www.Arabidopsis.org/), and the rice genome database (http://rice.plantbiology.msu.edu/, release 5.0). These sequences were then used to search *FRK*s from the Phytozome (http://phytozome.jgi.doe.gov/pz/portal.html) and GenBank for monocots - sorghum (*Sorghum bicolor*), maize (*Zea mays)*, dicots - potato (*Solanum tuberosum*), grape (*Vitis vinifera*) and tomato (*Solanum lycopersicum*). The sequences containing the pfkB domain with matches achieved similarity scores of 50.0, probability scores >50.0 and e-value <10 − 4, and were collected.

### Identification of *FRK* families from a BAC library

A BAC library was constructed for the haploid genome of *S. spontaneum* SES208, Ap85-441 (2n = 4× = 32), and contained 38,400 clones with an average insert size of 100 kb, resulting in 6× coverage of the whole genome [36].

As described by Yu et al. [45], BAC library screening was performed using the probe amplified with the primers shown in Additional file 9. The positive BAC clones were verified by PCR with the same primers and then sequenced according to the Sanger method by Takara (Dalian, China). Different haplotypes were selected. BAC DNAs were isolated using the PhasePrep™ TMBAC DNA kit (Sigma-Aldrich, NA0100-1KT), and the insert size of BAC clones was estimated by comparison with standard size markers using CHEF gel electrophoresis. The DNA of individual BAC clones was prepared with unique barcode using the DNA Library Prep Kit (NEB #E7645). The DNA-seq libraries were then pooled and sequenced with 150 bp, pair-end reads on Illumina HiSeq2500 at the Center for Genomics and Biotechnology in Fujian Agriculture and Forestry University. The BAC sequences were then assembled using SPAdes Genome Assembler v. 3.1.1(http://bioinf.spbau.ru/en/spades).

### Sequence analysis and phylogenetic tree

The BAC assembled contigs were annotated by DNA subway (http://dnasubway.iplantcollaborative.org/) with Sorghum and sugarcane ESTs from GenBank as references, the CDS sequences of the *FRK* genes were then translated into protein sequences by the EXPASy-translate tool (http://web.expasy.org/translate/). The putatively conserved domains of FRK proteins were detected by using BLASTp (http://blast.ncbi.nlm.nih.gov/Blast.cgi) and InterPro (http://www.ebi.ac.uk/interpro/scan.html). The isoelectric point and relative molecular mass of the proteins were predicted using ExPASy (http://web.expasy.org/compute_pi/). The exon-intron structures for the *FRK* genes were graphed using the online tool GSDS (http://gsds.cbi.pku.edu.cn/).

The amino acid sequences of *FRK* family members in 4 monocotyledons (*Zea mays, Sorghum bicolor, Oryza sativa,* and *Saccharum spontaneum*), and 4 dicotyledons (*Arabidopsis thaliana, Solanum lycopersicum, Solanum tuberosum* and *Vitis vinifera*) were used for phylogenetic tree analysis. The phylogenetic trees were constructed with the MEGA5.2.1 program, ClustalW alignment using default parameters.

We calculated pairwise distances between FRK alleles of *S. spontaneum* and their orthologs from Sorghum at synonymous sites (Ks) and non-synonymous sites (Ka) by using DnaSP (version 5.10.1 http://www.ub.edu/dnasp/) with default parameters. The FRK orthologs between *S. spontaneum* and sorghum were identified based on phylogenetic analysis and the protein sequence identities. The FRK genes of *S. spontaneum* and sorghum which phylogenetic distributed together and shared higher sequence similarity were considered to be the orthologs genes.

### Analysis of the co-expression profiling of fructokinases in *Saccharum* based on RNA-seq

5 μg total RNA of each sample were used for the construction of cDNA libraries. The cDNA libraries were prepared using Illumina ® TruSeq™ RNA Sample Preparation Kit (RS-122-2001(2), Illumina) according to the manufacturer’s protocol. The RNA-seq libraries were pooled and sequenced with 100 single reads on Illumina HiSeq2500 at the Center for Genomics and Biotechnology at the Fujian Agriculture and Forestry University.

Raw data were aligned to reference gene models using NOVOALIGN (http://www.novocraft.com/). NOVOALIGN reports multiple alignments for each read, however, the program discards alignments if the posterior alignment probability of the best alignment is less than 0.7 (http://www.novocraft.com/documentation/novoalign-2/novoalign-user-guide/novoalign-command-options/reporting-multiple-alignments-per-read/). Mappable reads were counted using htseq-count program (http://www-huber.embl.de/users/anders/HTSeq/doc/count.html) with union mode. To identify differentially expressed genes (DEGs), we applied edgeR package to calculate Reads Per Kilobase of exon model per Million mapped reads(RPKM) value and fold change (FC) for each gene [46]. Genes with |log2FC > 2|, FDR < 0.05 were considered as DEGs.

### Validation of RPKM values for *FRK* genes using qRT-PCR

RNA (≤1 μg) from each sample was reverse-transcribed to cDNA using the Reverse Transcriptase Kit (Takara) in a 20 μl reaction volume with 1 μl of random primers and 1 μl of mixed poly-dT primers (18–23 nt). The cDNA was diluted 1:7 in water for further qRT-PCR experiments.

The expression levels of *FRK* genes were validated using qRT-PCR in partial tissues of four sugarcane species. Gene-specific primer pairs (Additional file 9) were designed using the online PrimerQuest tool at Integrated DNA Technologies (IDT) (http://www.idtdna.com/Primerquest/Home/Index). Real-time qPCR were run on Multicolor Real-Time PCR Detection System (Bio-Rad). The real time PCR reaction program was 95 °C for 30s, 40 cycles at 95 °C for 5 s, followed by 60 °C for 30s, and PCR specificity was confirmed using a heat dissociation protocol from 65 to 95_C following the final cycle of the PCR. The glyceraldehyde-3-phosphate dehydrogenase gene (GAPDH) and Eukaryotic elongation factor 1a (eEF-1a) were selected as internal standards for normalization [47], and three replicates were run for each sample. The relative expression levels for each *FRK* gene in different tissues of three sugarcane species were calculated using the 2^-ΔΔCt^ method [48].

## Additional files

Additional file 1: BLAST results for ESTs of SsFRK in the NCBI database. (DOC 29 kb)
Additional file 2: Amino acid sequence pairwise comparisons (% similarity) between FRK gene family members in sugarcane. (DOC 31 kb)
Additional file 3: Comparison of gene expression levels of *SsFRK* gene family members by qRT-PCR and RNA-Seq. PM: premature; SES: *S. spontaneum* SES208; LA: *S. officinarum* LA Purple; IN:internode; LF, leaf. Using the data made a correlation analysis. (PPTX 119 kb)
Additional file 4: Heatmap of the expression levels of *SsFRK* gene family members in pre-mature stage and mature stage tissues. SR: *S. robustum* Molokai6081; SS: *S. spontaneum* SES208; SO: *S. officinarum* LA Purple; SH: hybrid cultivar ROC-22; IN: internode; LR: leaf roll; LF: leaf. Internnodes 3,9,15, internnodes 3,9,15, internodes 3,8,13 and internodes 3, 6, 9 were from *Saccharum officinarum* (LA Purple), ROC-22, *Saccharum robustum* (Molokai6081) and *Saccharum spontaneum* (SES208), respectively. (PPTX 156 kb)
Additional file 5: Heatmap of the expression levels of *SsFRK* gene family members under ABA treatment. SR: *S. robustum* Molokai6081; SS: *S. spontaneum* SES208; SO: *S. officinarum* LA Purple; SH: hybrid cultivar ROC-22; IN: internode; LR: leaf roll; LF: leaf. Internnodes 3,9,15, internnodes 3,9,15, internodes 3,8,13 and internodes 3, 6, 9 were from *Saccharum officinarum* (LA Purple), ROC-22, *Saccharum robustum* (Molokai6081) and *Saccharum spontaneum* (SES208), respectively. (PPTX 205 kb)
Additional file 6: Heatmap of the expression level of *SsFRK* gene family members under IAA treatment. SR: *S. robustum* Molokai6081; SS: *S. spontaneum* SES208; SO: *S. officinarum* LA Purple; SH: hybrid cultivar ROC-22; IN: internode; LR: leaf roll; LF: leaf. Internnodes 3,9,15, Internnodes 3,9,15, internodes 3,8,13 and internodes 3, 6, 9 were from *Saccharum officinarum* (LA Purple), ROC-22, *Saccharum robustum* (Molokai6081) and *Saccharum spontaneum* (SES208), respectively. (PPTX 194 kb)
Additional file 7: Heatmap of the expression levels of *SsFRK* gene family members under Et treatment. SR: *S. robustum* Molokai6081; SS: *S. spontaneum* SES208; SO: *S. officinarum* LA Purple; SH: hybrid cultivar ROC-22;IN: internode; LR: leaf roll; LF: leaf. Internnodes 3,9,15, internnodes 3,9,15, internodes 3,8,13 and internodes 3, 6, 9 were from *Saccharum officinarum* (LA Purple), ROC-22, *Saccharum robustum* (Molokai6081) and *Saccharum spontaneum* (SES208), respectively. (PPTX 193 kb)
Additional file 8: Heatmap of the expression levels of *SsFRK* gene family members under GA treatment. SR: *S. robustum* Molokai6081; SS: *S. spontaneum* SES208; SO: *S. officinarum* LA Purple; SH: hybrid cultivar ROC-22; IN: internode; LR: leaf roll; LF: leaf. Internnodes 3,9,15, Internnodes 3,9,15, internodes 3,8,13 and internodes 3, 6, 9 were from *Saccharum officinarum* (LA Purple), ROC-22, *Saccharum robustum* (Molokai6081) and *Saccharum spontaneum* (SES208), respectively. (PPTX 195 kb)
Additional file 9: The primers for qRT-PCR verification of five *SsFRK* in four *Saccharum* species. (DOC 29 kb)

## Abbreviations

ABA: Abscisic acid
ATP: Adenosine triphosphate
BACs: Bacterial artificial chromosomes
EST: Expressed sequence tags
Et: Ethephon
F6P: Fructose 6-phosphate
FRK: Fructokinases
Fru: Fructose
GA: Gibberellins
IAA: Auxin
LCA: The last common ancestor
Mya: Million years ago
PEG: Polyethylene glycol
RPKM: Reads Per Kilobases per Millionread
