# Supplementary material for: Evolution and expression of the fructokinase gene family in Saccharum
Source: BMC Genomics. 2017 Feb 21;18:197. doi: 10.1186/s12864-017-3535-7 (PMC5319016; doi:10.1186/s12864-017-3535-7)
Supplement: Additional file 1: — BLAST results for ESTs of SsFRK in the NCBI database. (DOC 29 kb) [file 12864_2017_3535_MOESM1_ESM.doc]

**Additional file 1: BLAST results for ESTs of *SsFRK*** from the NCBI database

| **Gene Name** | **The number of aligned EST** | **The Accession of aligned EST** |
| --- | --- | --- |
| ***SsFRK1*** | **14** | **CA162623.1 CA275688.1 CA157644.1 CA261051.1 CA108940.1 CA216906.1 CA100286.1 CA183452.1 CA076844.1 CA276482.1 CA246373.1 CA166173.1 CA270271.1 CA097328.1** |
| ***SsFRK2*** | **5** | **CA083414.1 CA090434.1 CA201686.1 CA201605.1 CA200395.1** |
| ***SsFRK3*** | **0** |  |
| ***SsFRK4*** | **2** | **CA086857.1 CA087119.1** |
| ***SsFRK5*** | **1** | **CA075518.1** |
| ***SsFRK6*** | **2** | **DV638085.1 CA111204.1** |
| ***SsFRK7*** | **3** | **CA166867.1 CA170603.1 CA130997.1** |
