# Supplementary material for: Evolution and expression of the fructokinase gene family in Saccharum
Source: BMC Genomics. 2017 Feb 21;18:197. doi: 10.1186/s12864-017-3535-7 (PMC5319016; doi:10.1186/s12864-017-3535-7)
Supplement: Additional file 2: — Amino acid sequence pairwise comparisons (% similarity) between FRK gene family members in sugarcane. (DOC 31 kb) [file 12864_2017_3535_MOESM2_ESM.doc]

**Additional file 2: Amino acid sequence pairwise comparisons (％ similarity ) of *FRK*** gene family members in sugarcane

|  | ***SsFRK1*** | ***SsFRK2*** | ***SsFRK3*** | ***SsFRK4*** | ***SsFRK5*** | ***SsFRK6*** | ***SsFRK7*** |
| --- | --- | --- | --- | --- | --- | --- | --- |
| ***SsFRK1*** |  |  |  |  |  |  |  |
| ***SsFRK2*** | 64% |  |  |  |  |  |  |
| ***SsFRK3*** | 33% | 32% |  |  |  |  |  |
| ***SsFRK4*** | 25% | - | - |  |  |  |  |
| ***SsFRK5*** | 33% | 31% | 39% | - |  |  |  |
| ***SsFRK6*** | - | - | - | - | - |  |  |
| ***SsFRK7*** | - | - | - | - | - | - |  |
