# Supplementary figures and images for: Evolution and expression of the fructokinase gene family in Saccharum
Source: BMC Genomics. 2017 Feb 21;18:197. doi: 10.1186/s12864-017-3535-7 (PMC5319016; doi:10.1186/s12864-017-3535-7)

## Slide 1
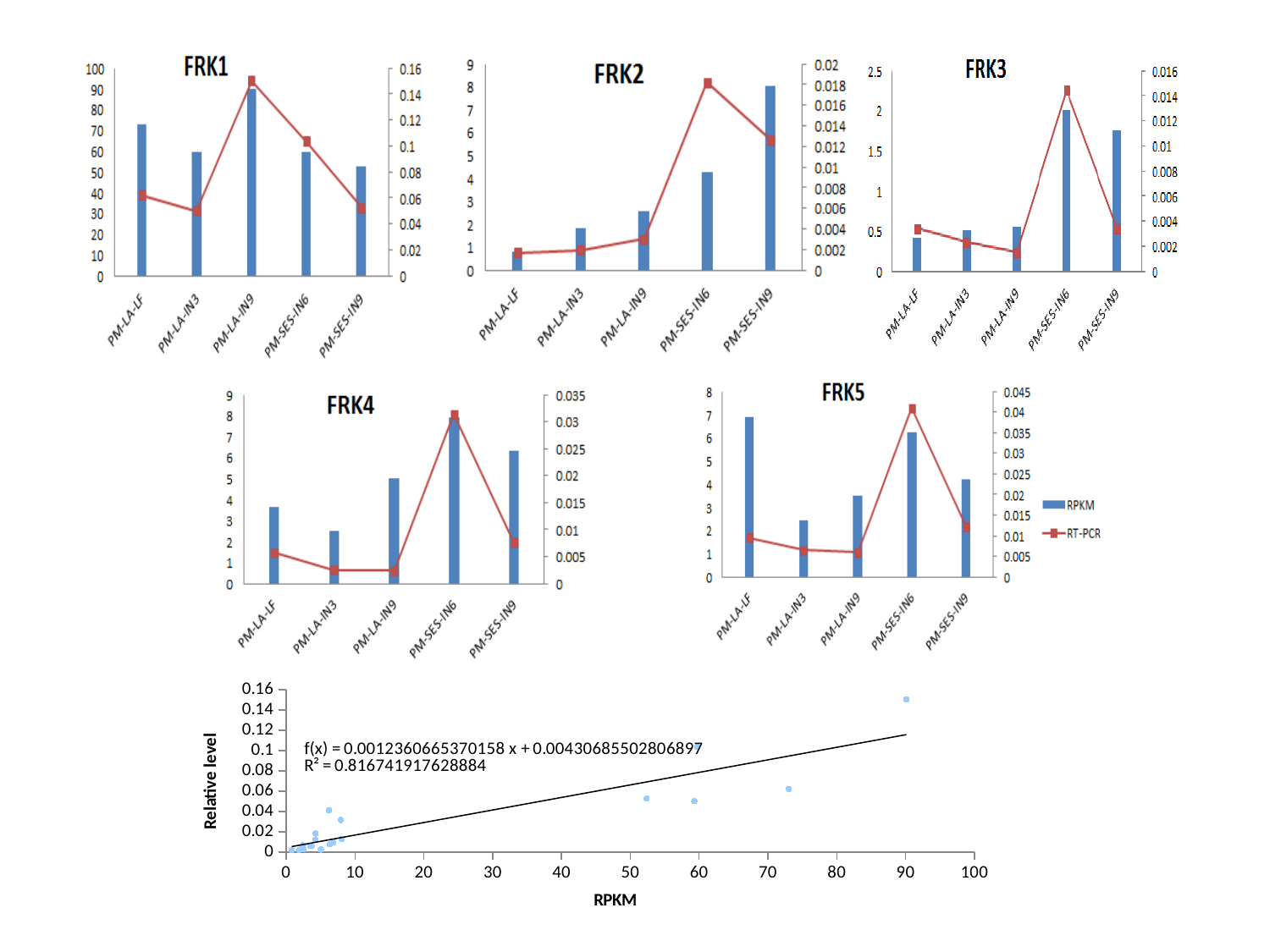

### Chart
| Category | |
|---|---|

Supplement: Additional file 3: — Comparison of gene expression levels of SsFRK gene family members by qRT-PCR and RNA-Seq. PM: premature; SES: S. spontaneum SES208; LA: S. officinarum LA Purple; IN:internode; LF, leaf. Using the data made a correlation analysis. (PPTX 119 kb) [file 12864_2017_3535_MOESM3_ESM.pptx]

## Slide 1
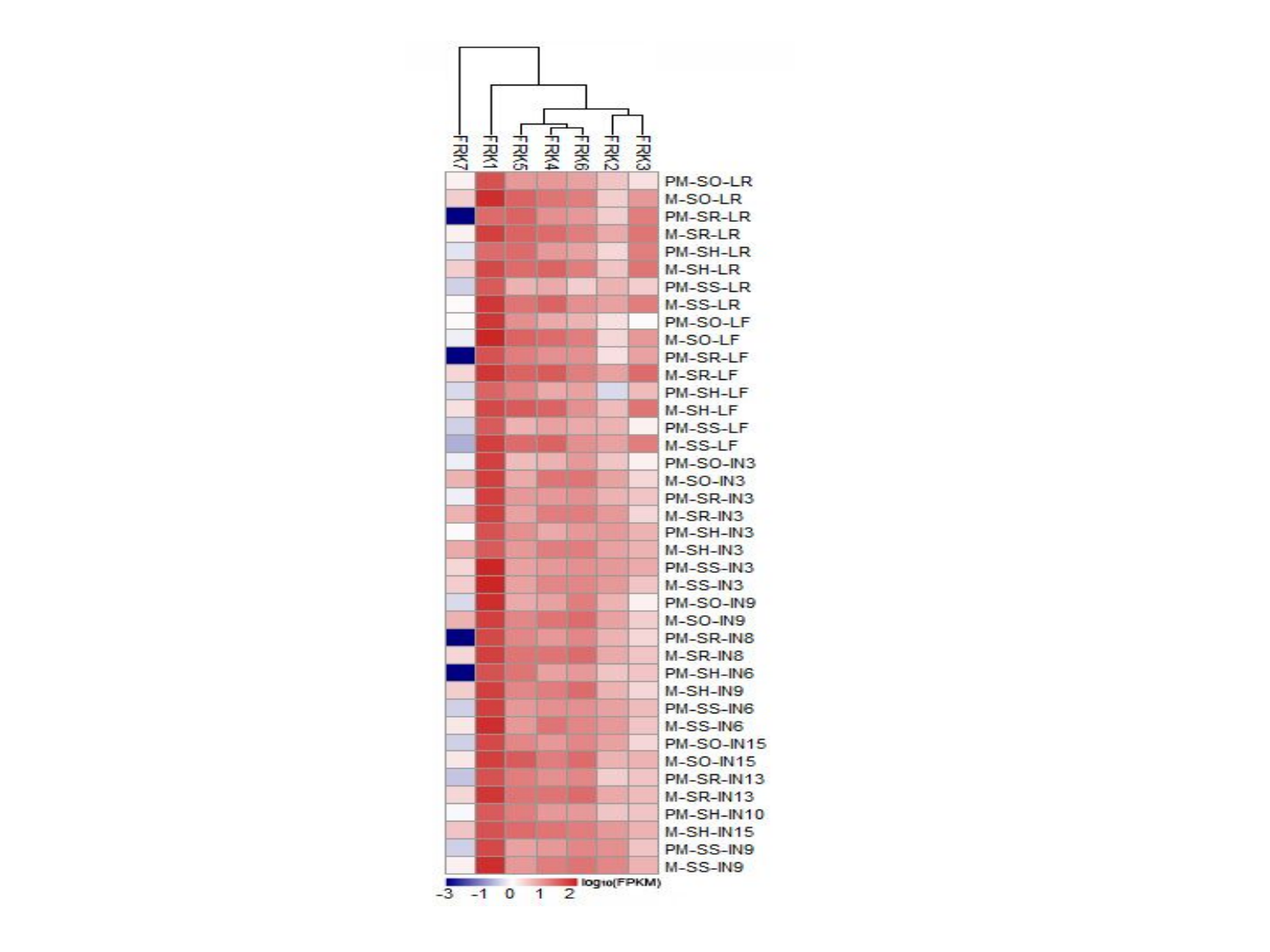

Supplement: Additional file 4: — Heatmap of the expression levels of SsFRK gene family members in pre-mature stage and mature stage tissues. SR: S. robustum Molokai6081; SS: S. spontaneum SES208; SO: S. officinarum LA Purple; SH: hybrid cultivar ROC-22; IN: internode; LR: leaf roll; LF: leaf. Internnodes 3,9,15, internnodes 3,9,15, internodes 3,8,13 and internodes 3, 6, 9 were from Saccharum officinarum (LA Purple), ROC-22, Saccharum robustum (Molokai6081) and Saccharum spontaneum (SES208), respectively. (PPTX 156 kb) [file 12864_2017_3535_MOESM4_ESM.pptx]

## Slide 1
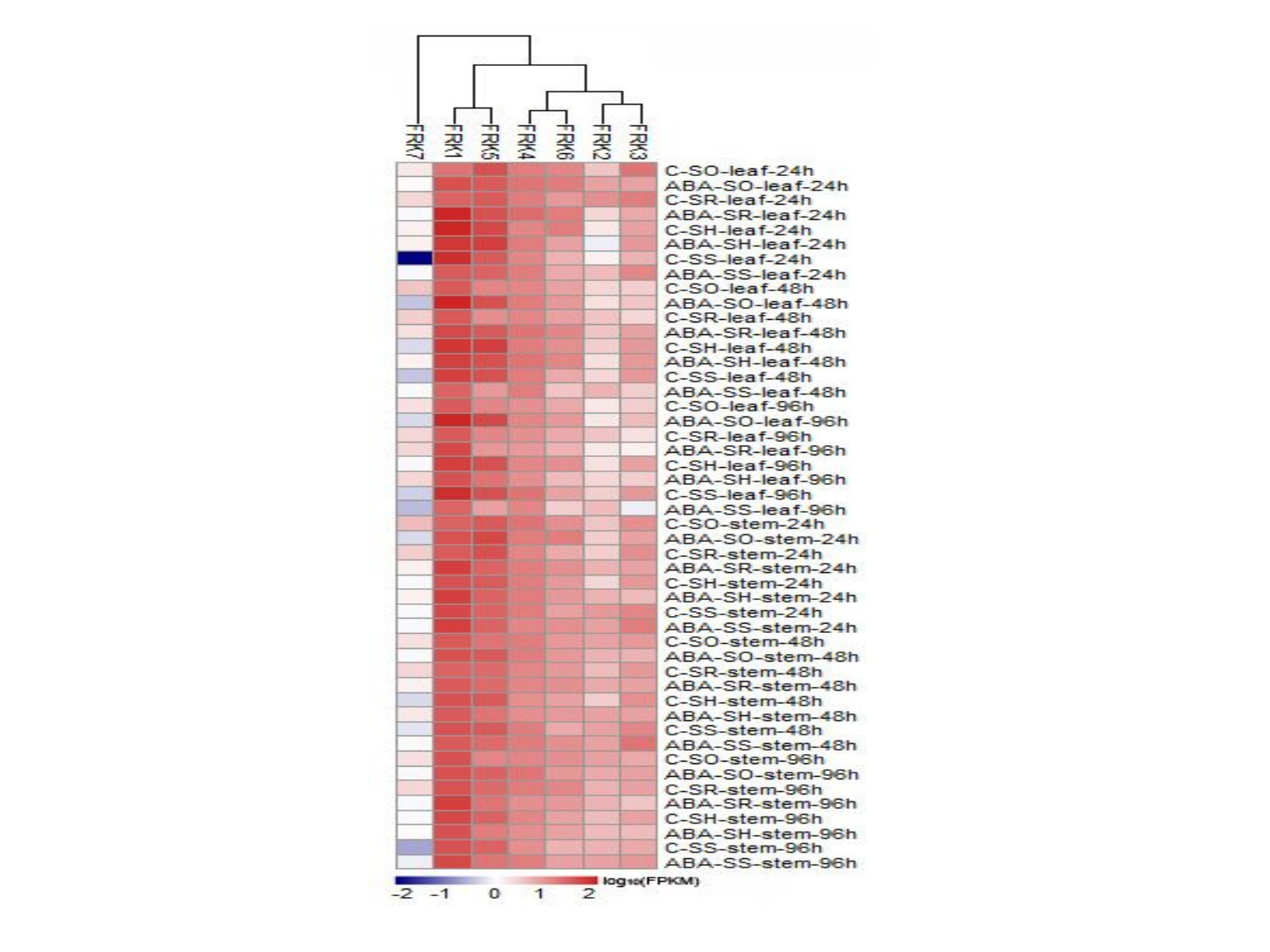

Supplement: Additional file 5: — Heatmap of the expression levels of SsFRK gene family members under ABA treatment. SR: S. robustum Molokai6081; SS: S. spontaneum SES208; SO: S. officinarum LA Purple; SH: hybrid cultivar ROC-22; IN: internode; LR: leaf roll; LF: leaf. Internnodes 3,9,15, internnodes 3,9,15, internodes 3,8,13 and internodes 3, 6, 9 were from Saccharum officinarum (LA Purple), ROC-22, Saccharum robustum (Molokai6081) and Saccharum spontaneum (SES208), respectively. (PPTX 205 kb) [file 12864_2017_3535_MOESM5_ESM.pptx]

## Slide 1
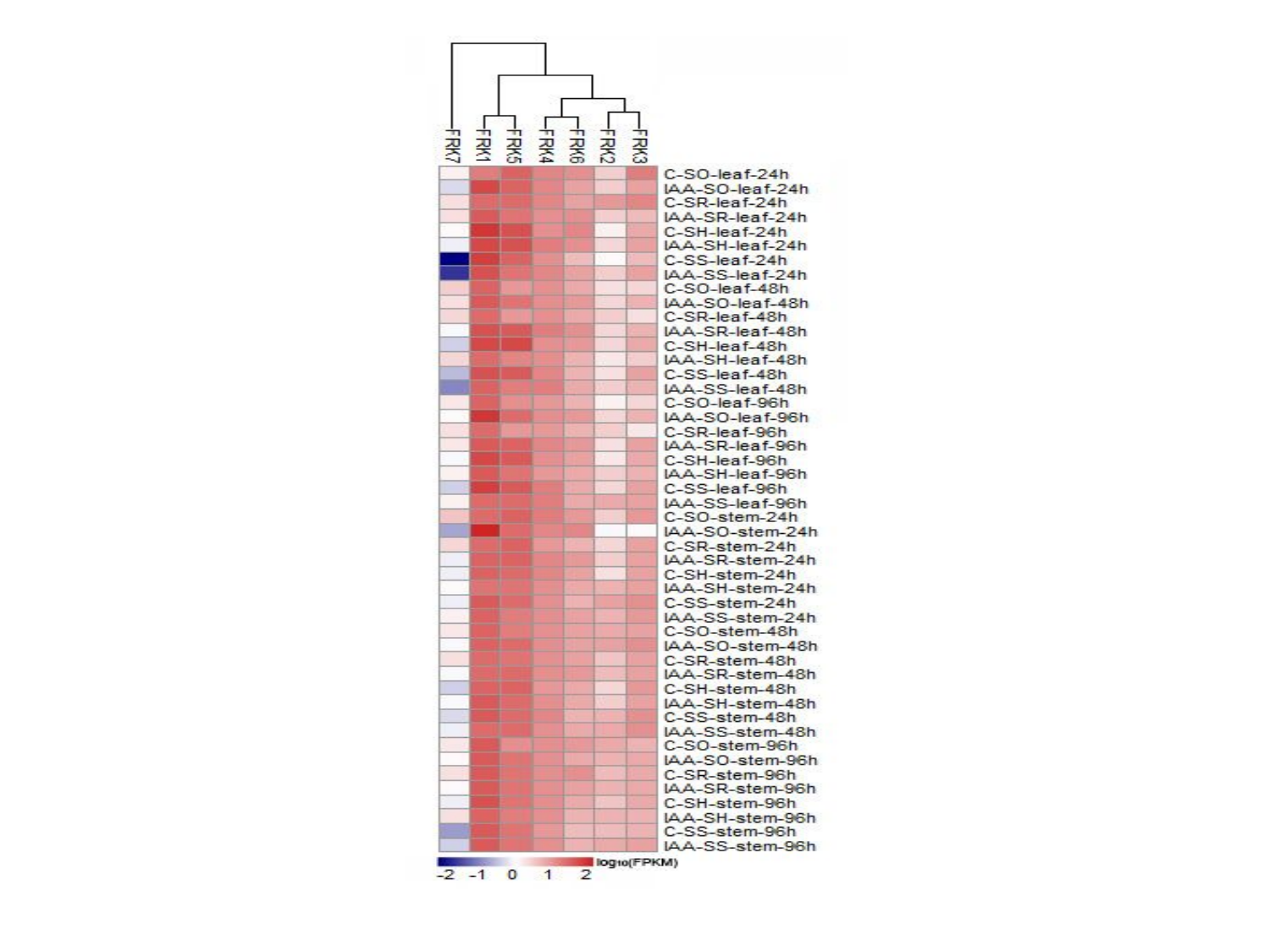

Supplement: Additional file 6: — Heatmap of the expression level of SsFRK gene family members under IAA treatment. SR: S. robustum Molokai6081; SS: S. spontaneum SES208; SO: S. officinarum LA Purple; SH: hybrid cultivar ROC-22; IN: internode; LR: leaf roll; LF: leaf. Internnodes 3,9,15, Internnodes 3,9,15, internodes 3,8,13 and internodes 3, 6, 9 were from Saccharum officinarum (LA Purple), ROC-22, Saccharum robustum (Molokai6081) and Saccharum spontaneum (SES208), respectively. (PPTX 194 kb) [file 12864_2017_3535_MOESM6_ESM.pptx]

## Slide 1
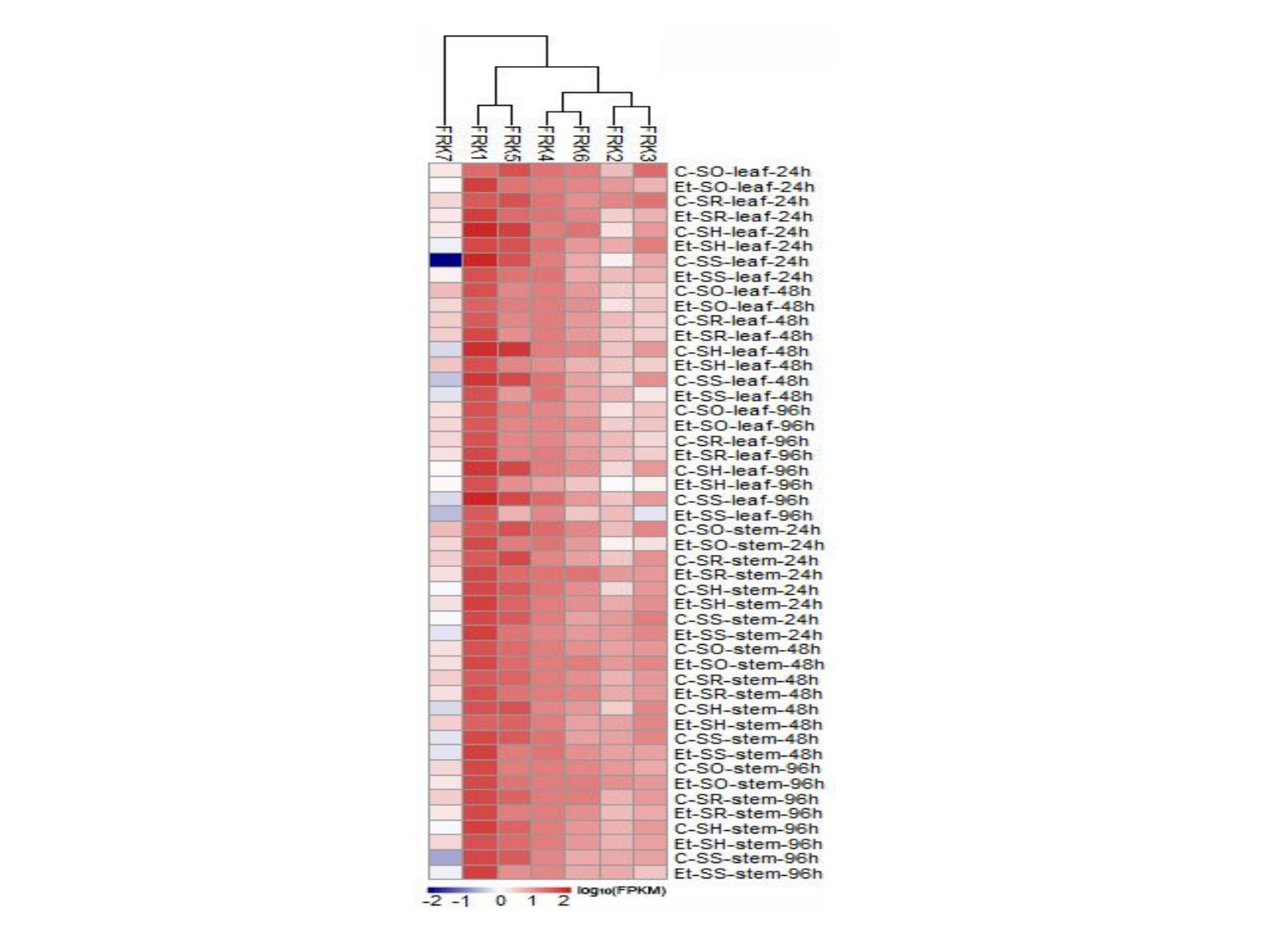

Supplement: Additional file 7: — Heatmap of the expression levels of SsFRK gene family members under Et treatment. SR: S. robustum Molokai6081; SS: S. spontaneum SES208; SO: S. officinarum LA Purple; SH: hybrid cultivar ROC-22;IN: internode; LR: leaf roll; LF: leaf. Internnodes 3,9,15, internnodes 3,9,15, internodes 3,8,13 and internodes 3, 6, 9 were from Saccharum officinarum (LA Purple), ROC-22, Saccharum robustum (Molokai6081) and Saccharum spontaneum (SES208), respectively. (PPTX 193 kb) [file 12864_2017_3535_MOESM7_ESM.pptx]

## Slide 1
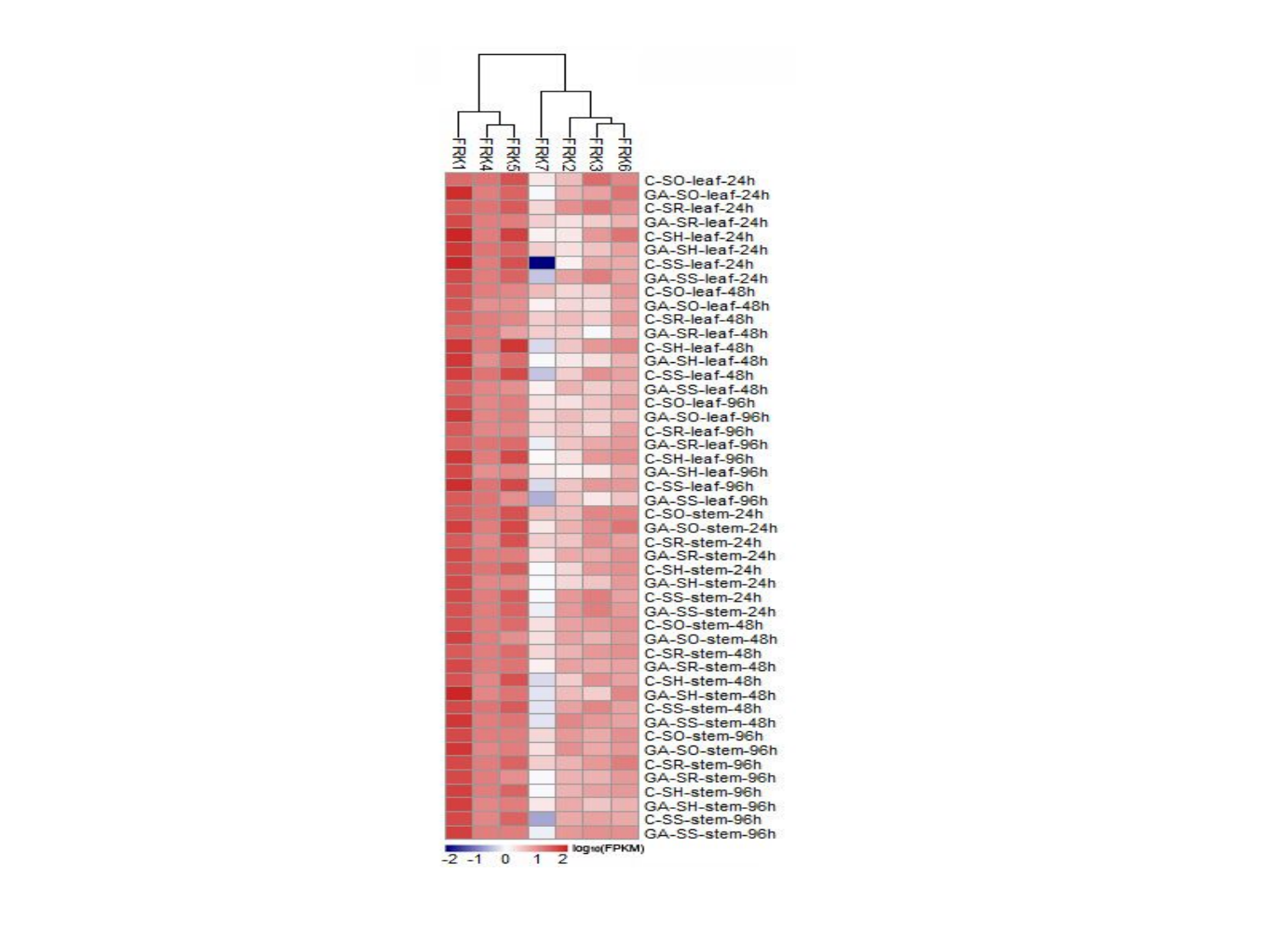

Supplement: Additional file 8: — Heatmap of the expression levels of SsFRK gene family members under GA treatment. SR: S. robustum Molokai6081; SS: S. spontaneum SES208; SO: S. officinarum LA Purple; SH: hybrid cultivar ROC-22; IN: internode; LR: leaf roll; LF: leaf. Internnodes 3,9,15, Internnodes 3,9,15, internodes 3,8,13 and internodes 3, 6, 9 were from Saccharum officinarum (LA Purple), ROC-22, Saccharum robustum (Molokai6081) and Saccharum spontaneum (SES208), respectively. (PPTX 195 kb) [file 12864_2017_3535_MOESM8_ESM.pptx]
