# Supplementary material for: Evolution and expression of the fructokinase gene family in Saccharum
Source: BMC Genomics. 2017 Feb 21;18:197. doi: 10.1186/s12864-017-3535-7 (PMC5319016; doi:10.1186/s12864-017-3535-7)
Supplement: Additional file 9: — The primers for qRT-PCR verification of five SsFRK in four Saccharum species. (DOC 29 kb) [file 12864_2017_3535_MOESM9_ESM.doc]

**Additional file 9: Primers for qRT-PCR verification of five *SsFRKs*** from four Saccharum species

| **Gene name** | **Forward** | **Reverse** |
| --- | --- | --- |
| **SsFRK1** | **CTCAGTGGAGGATGATGTTGTC** | **CATGGAAATCCCTGGCATAGT** |
| **SsFRK2** | **ACGCGCAGTTCAAACAAAG** | **ACAGTTGGGACGAAGTCAATTA** |
| **SsFRK3** | **TCAGAGGAGGAGAAGGATAAGG** | **CCCTCCATCATCATCACTAGATTC** |
| **SsFRK4** | **GACACAGTTGGATGTGGAGATAG** | **CTGCATTTGCTAGGGTTAATGTG** |
| **SsFRK5** | **GGACAGAGGATGCACTCATAAC** | **CCTAATAGCAGCAGCCACAATA** |
